# *Arabidopsis* histone demethylases LDL1 and LDL2 control primary seed dormancy by regulating *DELAY OF GERMINATION 1* and ABA signaling-related genes

**DOI:** 10.3389/fpls.2015.00159

**Published:** 2015-03-17

**Authors:** Minglei Zhao, Songguang Yang, Xuncheng Liu, Keqiang Wu

**Affiliations:** ^1^Key Laboratory of South China Agricultural Plant Molecular Analysis and Genetic Improvement, South China Botanical Garden, Chinese Academy of SciencesGuangzhou, China; ^2^University of Chinese Academy of SciencesBeijing, China; ^3^Institute of Plant Biology, National Taiwan UniversityTaipei, Taiwan

**Keywords:** histone demethylase, seed dormancy, ABA, gene expression, DOG1

## Abstract

Seed dormancy controls germination and plays a critical role in regulating the beginning of the life cycle of plants. Seed dormancy is established and maintained during seed maturation and is gradually broken during dry storage (after-ripening). The plant hormone abscisic acid (ABA) and DELAY OF GERMINATION1 (DOG1) protein are essential regulators of seed dormancy. Recent studies revealed that chromatin modifications are also involved in the transcription regulation of seed dormancy. Here, we showed that two *Arabidopsis* histone demethylases, LYSINESPECIFIC DEMETHYLASE LIKE 1 and 2 (LDL1 and LDL2) act redundantly in repressing of seed dormancy. *LDL1* and *LDL2* are highly expressed in the early silique developing stage. The *ldl1 ldl2* double mutant displays increased seed dormancy, whereas overexpression of *LDL1* or *LDL2* in *Arabidopsis* causes reduced dormancy. Furthermore, we showed that *LDL1* and *LDL2* repress the expression of seed dormancy-related genes, including *DOG1*, *ABA2* and *ABI3* during seed dormancy establishment. Furthermore, genetic analysis revealed that the repression of seed dormancy by LDL1 and LDL2 requires *DOG1*, *ABA2*, and *ABI3*. Taken together, our findings revealed that LDL1 and LDL2 play an essential role in seed dormancy.

## Introduction

Accurate timing of seed germination requires a reliable control mechanism. Seed dormancy is a major factor in this control, which refers to the seed property that incapacitates seed germination even under optimal conditions (Hilhorst, 2007). Seed dormancy prevents or delays the germination of maturated seed until conditions are favorable for starting a new life cycle. Seed dormancy is established during seed maturation, and dormancy has been shown to be imposed by the embryo, testa, endosperm or combinations of these tissues (Kim et al., 2013). Seed dormancy can be broken after a period of seed after-ripening or on seed stratification, that is, exposure to cold and moist conditions.

Diverse endogenous and environmental factors including phytohormones, nutrients, temperature and light affect seed dormancy through different pathways (Finkelstein et al., 2008). Emerging evidences have shown that abscisic acid (ABA) plays a critical role in the establishment and maintenance of seed dormancy (Finch-Savage and Leubner-Metzger, 2006; Holdsworth et al., 2008; North et al., 2010). Genetic studies demonstrate that loss-of-function mutants of ABA biosynthesis genes in *Arabidopsis* such as *ABA1*, *ABA2*, *ABA3*, and *NCED6/9* show reduced seed dormancy (Koornneef et al., 1982; Giraudat et al., 1992; Leon-Kloosterziel et al., 1996; Lefebvre et al., 2006; Okamoto et al., 2006), whereas loss-of-function mutants of ABA catabolism genes such as *CYP707A1*, *CYP707A2*, and *CYP707A3* display enhanced seed dormancy, supporting an essential role of ABA for seed dormancy (Kushiro et al., 2004; Okamoto et al., 2006; Finkelstein et al., 2008; Holdsworth et al., 2008) Furthermore, many components involved in ABA signaling transduction also influence the degree of seed dormancy. *abi1-1*, a gain-of-function mutant of *ABI1* encoding a member of group A protein phosphatase 2Cs (group A PP2Cs), shows reduced seed dormancy (Koornneef et al., 1984; Finkelstein, 1994). Furthermore, ABI3, a seed-specific B3 domain-containing transcription factor, was revealed to be necessary for the establishment of seed dormancy (Sugliani et al., 2009).

Seed dormancy is a typical quantitative trait. In *Arabidopsis thaliana*, the Columbia-0 (Col-0) and Landsberg erecta (Ler) ecotypes display relatively weak seed dormancy, whereas the Cape Verde Islands (Cvi) ecotype shows strong seed dormancy (Alonso-Blanco et al., 2003). Analysis of quantitative trait loci (QTL) using recombinant inbred lines between Ler and Cvi identified several *Delay of Germination* (*DOG*) genes (Alonso-Blanco et al., 2003). Among them, *DOG1* is the master regulator, which is only expressed in seeds and its expression level is increased during seed maturation (Bentsink et al., 2006). In freshly harvested seeds, the time required for dormancy release is determined by the DOG1 protein level. Furthermore, it was proposed that DOG1 acts independent of ABA signaling (Nakabayashi et al., 2012). Collectively, these findings suggested that both ABA and DOG1 are required for seed dormancy.

Recent studies suggested an involvement of epigenetic regulators in seed dormancy and germination (Liu et al., 2014). REDUCED DORMANCY 4 (RDO4)/HISTONE MONOUBIQUITINATION 1 (HUB1) and its homolog HUB2 influence seed dormancy through ubiquitination of H2B, leading to changes in histone H3 methylation of the seed dormancy-related genes (Liu et al., 2007). FERTILIZATION INDEPENDENT ENDOSPERM (FIE), an essential component of the polycomb repressive complex 2 (PRC2,) reduces seed dormancy by repressing *ABI3*, ABA/GA signaling factors and *DOG1* expression (Bouyer et al., 2011). Furthermore, the histone methyltransferases *KRYPTONITE (KYP)/SUVH4* and *SUVH5* repress *DOG1* and *ABI3* expression during seed maturation (Zheng et al., 2012). More recently, it was reported that *histone deacetylase9* (*hda9*) mutants display reduced seed dormancy. Transcriptome analysis revealed that *HDA9* repressed the expression of photosynthesis and photoautotrophic growth-related genes in dry seeds (van Zanten et al., 2014). Collectively, these findings revealed that chromatin modifications, including histone acetylation, methylation and ubiquitination are required for the transcriptional regulation of seed dormancy.

LDL1 and LDL2, two *Arabidopsis* homolog of the human LYSINESPECIFIC DEMETHYLASE 1 (LSD1), have been reported to play an important role in control of flowering. LDL1 and LDL2 reduce the histone H3-Lys 4 methylation levels in chromatin of the floral repressors *FLOWERING LOCUS C* (*FLC*) and *FWA*. Loss-of-function mutants of *LDL1* and *LDL2* show increased expression levels of *FLC* and *FWA* and late flowering phenotype (Jiang et al., 2007). Here in our present work, we showed that LDL1 and LDL2 act redundantly in repressing of seed dormancy. The *ldl1* or *ldl2* single mutant do not change the seed dormancy level, while the *ldl1 ldl2* double mutants display strong increased seed dormancy, what's more, overexpression of *LDL1* or *LDL2* in *Arabidopsis* causes reduced seed dormancy. Furthermore, LDL1 and LDL2 repress the expression levels of dormancy-related genes, including *DOG1*, *ABA2*, and *ABI3* in maturating seeds. Our studies suggest that LDL1 and LDL2 play an essential role in regulating primary seed dormancy by mediating *DOG1* expression and ABA signaling pathway.

## Results

### Subcellular localization of LDL1 and LDL2

**Figure 1:**
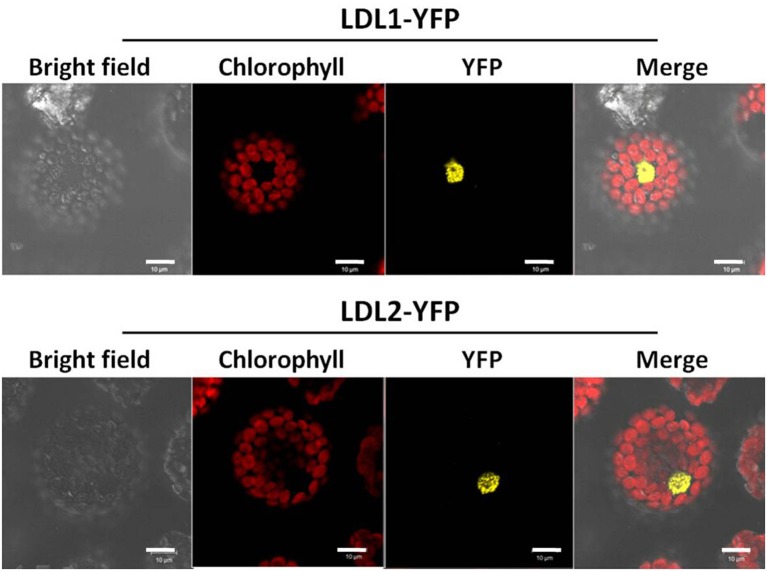
**Subcellular localization analysis of LDL1 and LDL2**. Constructs of LDL1-YFP and LDL2-YFP were transfected into *Arabidopsis* protoplasts. The fluorescence signal was detected with a laser scanning confocal microscope. YFP indicates fluorescence of YFP, and the red color indicates the auto-fluorescence of chlorophyll. The length of the bar is 10 μm.

To investigate the subcellular localization of LDL1 and LDL2 proteins, full-length coding sequences of *LDL1* and *LDL2* fused with yellow fluorescent protein (YFP) were delivered to the *Arabidopsis* protoplasts. LDL1-YFP and LDL2-YFP proteins were observed to be localized in the nucleus of the protoplast (Figure 1).

### Expression patterns of *LDL1* and *LDL2* during seed maturation

**Figure 2:**
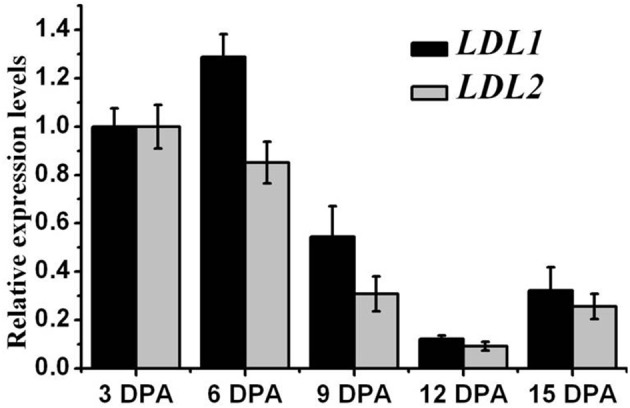
**Expression patterns of *LDL1* and *LDL2* during seed maturation**. DPA, days post-anthesis. *UBQ10* was used as an internal control. At least three biological replicates were conducted. The average (±SD) values are shown.

To characterize the roles of *LDL1* and *LDL2* in plant development, we first checked their expression patterns through the public *Arabidopsis* microarray database (http://www.bar.utoronto.ca/efp/cgi-bin/efpWeb.cgi). *LDL1* and *LDL2* are highly expressed in seed development stage (Supplemental Figure 1). We further investigated the expression patterns of *LDL1* and *LDL2* by quantitative RT-PCR assays. Consistently, relatively higher expression levels of *LDL1* and *LDL2* were detected at 3 and 6 DPA (days post-anthesis) siliques, whereas the expression levels of *LDL1* and *LDL2* were gradually decreased from 9 DPA (Figure 2). Our data reveal a possible role of *LDL1* and *LDL2* in seed development.

### Mutations in *LDL1* and *LDL2* increase primary seed dormancy

**Figure 3:**
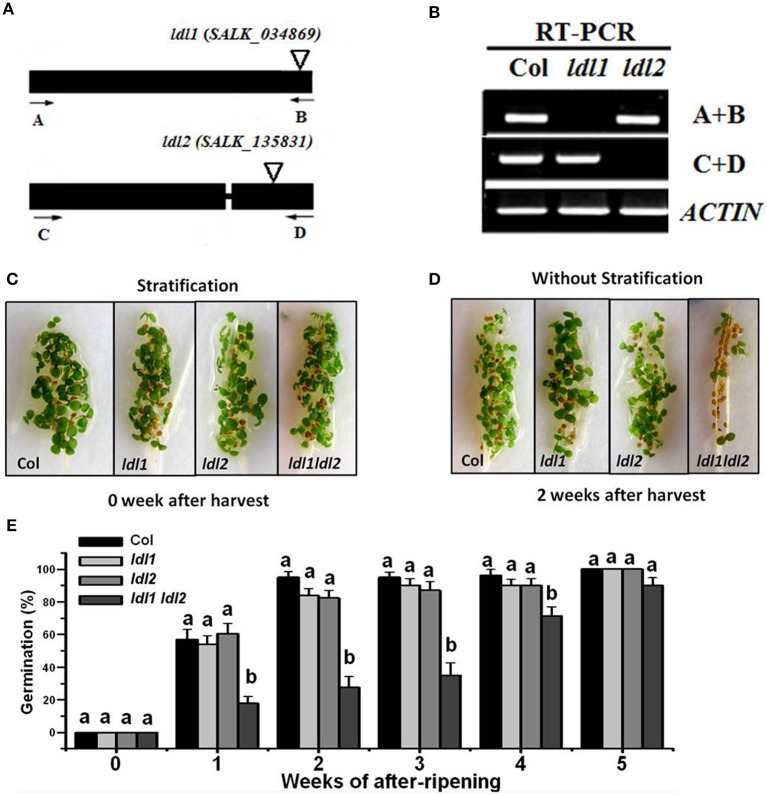
***ldl1 ldl2* double mutant shows increased primary seed dormancy**. **(A)** The gene structures and T-DNA insertion sites of *ldl1* and *ldl2*. The black boxes, lines and triangles indicate exons, introns and T-DNA insertions, respectively. **(B)** RT-PCR analysis of the expression levels of *LDL1* and *LDL2* in *ldl1* and *ldl2* alleles. Leaves of 15-day-old plants were harvested for analysis. The sites of the primer pairs used for RT-PCR analysis were indicated with arrows in **(A)**. *ACTIN* was used as a loading control. **(C)** Visualization of seed germination of *ldl1*, *ldl2*, and *ldl1 ldl2* siliques 0 week after harvest. The siliques were stratified and sown on water-saturated filter papers for 7 d. **(D)** Visualization of seed germination of *ldl1*, *ldl2*, and *ldl1 ldl2* siliques 2 week after harvest. The siliques were sown on water-saturated filter papers for 7 d without stratification. **(E)** Quantification of germination rates of non-stratified seeds of *ldl1*, *ldl2*, and *ldl1 ldl2* with different periods of after-ripening. The germination rates were scored 5 d after plated on 1/2 MS medium. The average (±SD) values are shown. One-Way ANOVA (Tukey-Kramer test) analysis was performed, and statistically significant differences (*P* < 0.01) were indicated by different lowercase letters (a, b). Equivalent means have the same letter; different letters indicate statistically significant differences.

To study the function of *LDL1* and *LDL2*, two T-DNA insertion mutants, *ldl1* (SALK_034869) and *ldl2* (SALK_135831) were analyzed (Figure 3A). RT-PCR analyses showed that the transcripts of the full-length *LDL1* and *LDL2* were disrupted in *ldl1* and *ldl2* mutants (Figure 3B). The *ldl1 ldl2* double mutant was also generated by genetic crossing. The freshly harvested seeds of Col, *ldl1*, *ldl2*, and *ldl1 ldl2* with stratification treatment were all well germinated (Figure 3C). Then, the germination rates of the *ldl1*, *ldl2*, and *ld1 ldl2* mutants were scored after stored in dry conditions for different times. After 1–5 weeks of storage, the *ldl1* and *ldl2* single mutants display no significant effect on seed germination, however, the *ldl1 ldl2* double mutant shows a significant decrease of seed germination (Figures 3D,E), suggesting that LDL1 and LDL2 may be functionally redundant in repressing of seed dormancy.

### Overexpression of LDL1 or LDL2 reduces primary seed dormancy

**Figure 4:**
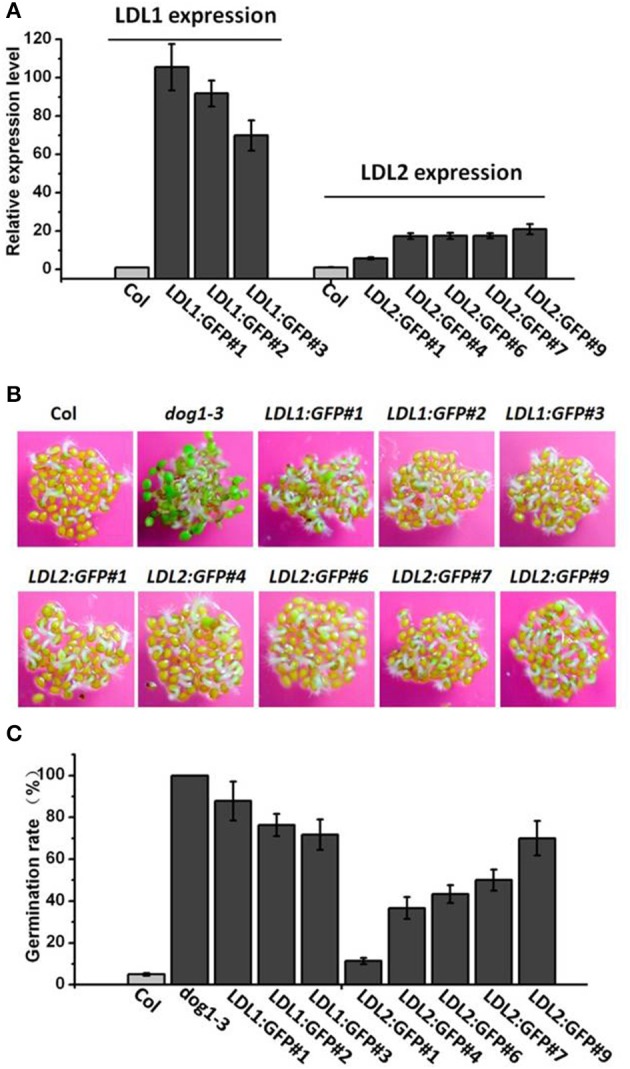
**Overexpression of *LDL1* or *LDL2* in *Arabidopsis* reduces seed dormancy**. **(A)** Quantitative analysis of the expression levels of *LDL1* and *LDL2* in the GFP-tagged transgenic lines. Leaves of 15-day-old plants were harvested for analysis. UBQ10 was used as an internal control. **(B)** Visualization of seed germination of *LDL1* and *LDL2* overexpression lines. **(C)** Quantification of germination rates of with 4 days' after-ripening after 3 days on 1/2 MS. The average (±SD) values are shown. The seeds of 4 d after-ripening were plated on 1/2 MS medium for 3 d.

To further investigate the effect of LD1 and LDL2 on seed dormancy, we generated *LDL1* and *LDL2* overexpression plants. The coding regions of *LDL1* and *LDL2* were introduced to the vector pCAMBIA-1302 under the control of the Cauliflower Mosaic Virus (CaMV) 35S promoter, separately. Increased expression levels of *LDL1* and *LDL2* were detected in the transgenic plants (Figure 4A). The *LDL1* and *LDL2* overexpression lines display enhanced seed germination compared with the wild-type (Figures 4B,C), confirming a negative role of LDL1 and LDL2 in primary seed dormancy. Collectively, our findings suggest that LDL1 and LDL2 may function redundantly in the repression of primary seed dormancy in *Arabidopsis*.

### *ldl1 ldl2* mutant shows increased ABA sensitivity during seed germination

**Figure 5:**
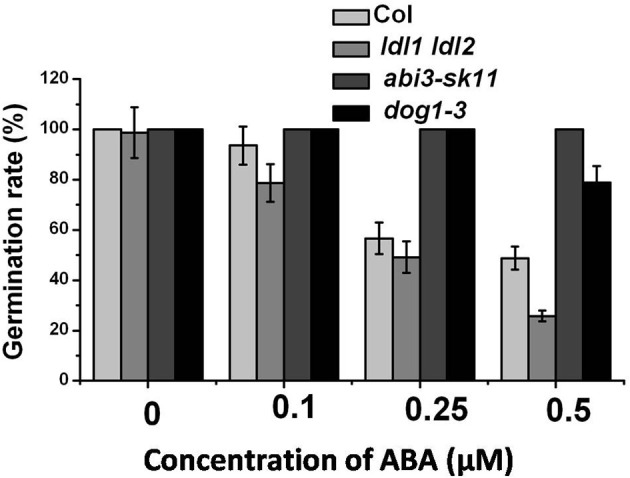
***ldl1 ldl2* increases ABA sensitivity during seed germination**. The seeds of 10 d after-ripening were stratified and plated on 1/2 MS medium supplemented with various concentrations of ABA. The germination rates were scored 3 d after plating. The average (±SD) values are shown.

Previous works reported that the mutants with a deep degree of seed dormancy such as the histone methyltransferase mutant *kyp/suvh4* are hypersensitive to ABA. We further tested the sensitivity of *ldl1 ldl2* mutant to ABA during germination. The ABA insensitive mutants, *abi3-sk11* (Park et al., 2011) and *dog1-3*, were also analyzed. Here *dog1-3* used in our study shown different to *dog1*, which is a sensitive ABA mutant (Bentsink et al., 2006), this might be explained by two *Arabidopsis* accessions were used, *dog1* mutant is in L*er* background, whereas *dog1-3* is in Columbia background. Opposite to *abi3-sk11* and *dog1-3*, the *ldl1 ldl2* seeds plated on 1/2 MS medium supplemented with ABA display enhanced sensitivity compared with the wild-type (Figure 5), suggesting that LDL1 and LDL2 might be involved in ABA signaling pathway.

### LDL1 and LDL2 repress *ABA2*, *ABI3* and *ABI5* expression

**Figure 6:**
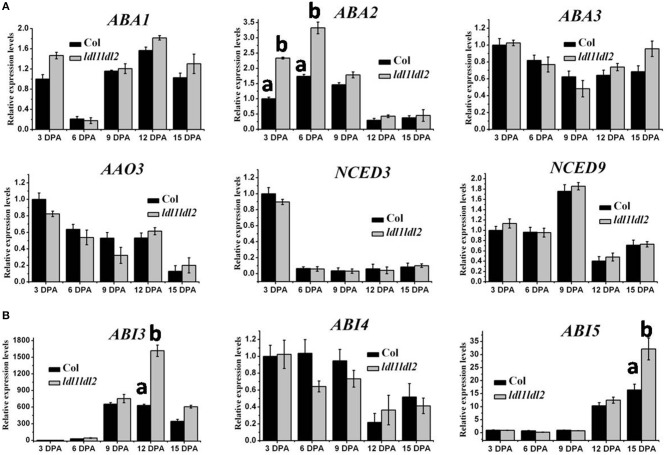
**Expression levels of ABA biosynthesis and signal transduction-related genes in *ldl1 ldl2* during seed maturation**. **(A)** Transcription analysis of genes related to ABA biosynthesis in Col and *ldl1 ldl2*. **(B)** Transcription analysis of genes related to ABA signal transduction in Col and *ldl1 ldl2. UBQ10* was used as an internal control. At least three biological replicates were conducted. The average (±SD) values are shown. One-Way ANOVA (Tukey-Kramer test) analysis was performed, and statistically significant differences (*P* < 0.01) were indicated by different lowercase letters (a, b). Equivalent means have the same letter; different letters indicate statistically significant differences.

Previous reports demonstrated that the ABA biosynthesis genes, *ABA1*, *ABA2*, *ABA3*, *AAO3*, *NCED3*, and *NCED9*, and the ABA signal transduction-related factors, *ABI3*, *ABI4*, and *ABI5*, play key roles in seed dormancy (Seo et al., 2000, 2006; Iuchi et al., 2001; Lopez-Molina et al., 2001; Xiong et al., 2001; Gonzalez-Guzman et al., 2002; Sugliani et al., 2010). We further detected the expression levels of these genes in *ldl1 ldl2* mutants. Among the tested ABA biosynthesis genes, the transcript of *ABA2* was significantly increased at 3 DPA and 6 DPA in *ldl1 ldl2* mutants (Figure 6A). Notably, the expression levels of the ABA signaling transduction components, *ABI3* and *ABI5*, were significantly up-regulated at 12 and 15 DPA, respectively, in *ldl1 ldl2* mutants (Figure 6B). Taken together, the above results indicated that *LDL1 and LDL2* may decrease primary seed dormancy by repressing ABA biosynthesis and signaling transduction related gene expression.

### LDL1 and LDL2 repress *DOG1* expression

**Figure 7:**
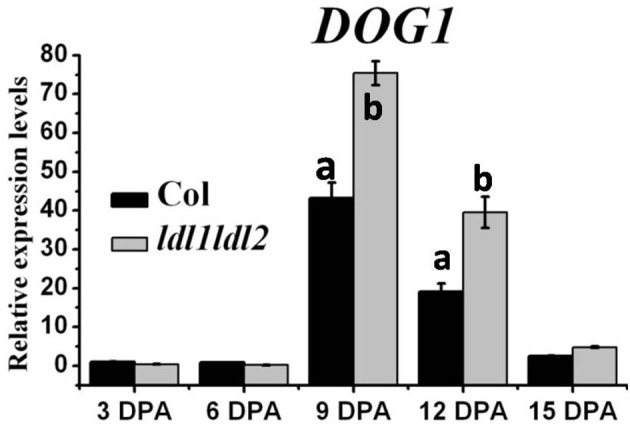
**Transcription analysis of *DOG1* expression in Col and *ldl1 ldl2* during seed maturation**. *UBQ10* was used as an internal control. At least three biological replicates were conducted. The average (±SD) values are shown. One-Way ANOVA (Tukey-Kramer test) analysis was performed, and statistically significant differences (*P* < 0.01) were indicated by different lowercase letters (a, b). Equivalent means have the same letter; different letters indicate statistically significant differences.

Previous work has demonstrated that DOG1 is the master regulator of seed dormancy which is only expressed in seed and its expression level increases during seed maturation (Bentsink et al., 2006). We further detect the expression level of *DOG1* in *ldl1* *ldl2* mutant. A previous report revealed that the transcription levels of *DOG1* reach the peak at around 16 DPA (Nakabayashi et al., 2012). In present work, the *DOG1* expression accumulates the highest at 9 DPA (Figure 7), this may due to a different maturation speed of siliques under the specific environmental conditions in which the plants were growing. In present work, the seed development from pollination until fully mature ripe seeds needs 15 days. The transcription of *DOG1* was significantly enhanced in *ldl1 ldl2* compared with the wild-type at 9 and 12 DPA (Figure 7).

### Genetic relationship of *LDL1* and *LDL2* with *DOG1, ABA2* and *ABI3*

**Figure 8:**
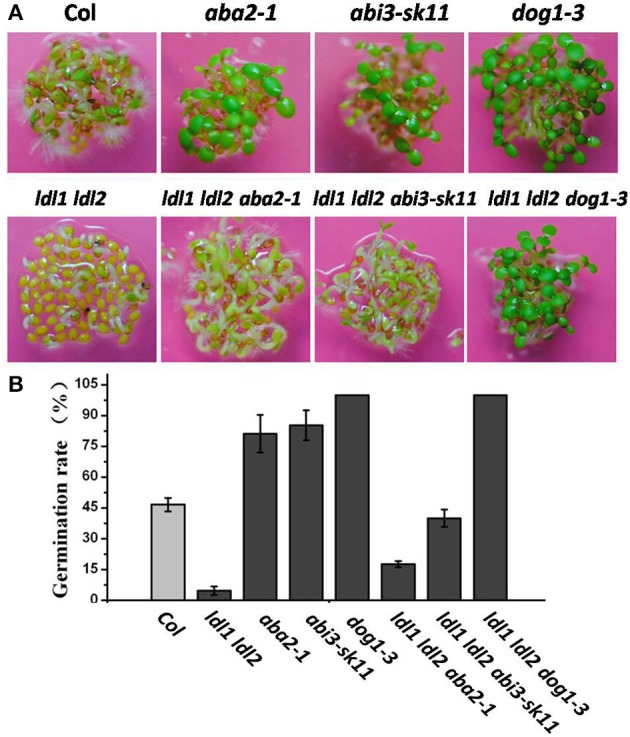
**Genetic analysis of the seed dormancy levels of *ldl1 ldl2* in *dog1-3*, *abi3-sk11* or *aba2-1* backgrounds**. **(A)** Visualization of seed germination of the mutants. **(B)** Quantification of the germination rates of the mutants. The average (±SD) values are shown. The seeds of 10 d after-ripening were used for analysis. The germination rates were scored 3 d after plated on 1/2 MS medium.

Elevated *ABA2*, *ABI3* and *DOG1* expression in *ldl1 ldl2* mutants during seed maturation prompted us to analyze the genetic relationship between *LDL1*,*LDL2* and *ABA2*, *ABI3* and *DOG1*. We crossed *ldl1 ldl2* with *dog1-3*, *aba2-1* and *abi3-sk11* mutants, respectively. As results, similar to *dog1-3*, *ldl1 ldl2 dog1-3* seeds were completely non-dormant (Figures 8A,B). Furthermore, the triple mutants *ldl1 ldl2 aba2-1* and *ldl1 ldl2 abi3-sk11* show an increase of germination rate compared with *ldl1 ldl2* mutants, respectively (Figures 8A,B). These data suggested that *DOG1*, *ABA2* and *ABI3* are required for LDL1 and LDL2-mediated repressing of seed dormancy.

## Discussion

In present work, we provided evidences that histone demethylases LDL1 and LDL2 are required for seed dormancy in *Arabidopsis*. Mutation in *LDL1* or *LDL2* do not change seed dormancy level, whereas *ldl1 ldl2* double mutant displays strong increased seed dormancy. In contrast, overexpression of *LDL1* or *LDL2* in *Arabidopsis* strongly decreases seed dormancy level. Taken together, our findings indicated that LDL1 and LDL2 play an essential role in seed dormancy.

ABA has been proved to play a critical role in establishment of seed dormancy. During seed maturation, the expression levels of the genes involved in ABA biosynthesis are increased and ABA signaling responses are enhanced (Xiong and Zhu, 2003). Consistently, loss-of-function mutants of ABA biosynthesis and signaling transduction components, such as *ABA2*, a key ABA biosynthetic gene, and *ABI3* which encodes a seed-specific B3 domain-containing DNA binding protein, show reduced primary seed dormancy levels in *Arabidopsis* (Lopez-Molina et al., 2002). In this study, loss-of-function and gain-of-function analysis revealed a negative role of LDL1 and LDL2 in primary seed dormancy. Increased ABA sensitivity of *ldl1 ldl2* seeds during germination indicates that LDL1 and LDL2 may regulate seed dormancy through ABA signaling pathway. Transcriptional analysis showed that the expression levels of *ABA2* and *ABI3* are increased in *ldl1 ldl2*, suggesting that LDL1 and LDL2 may control seed dormancy level by regulating both the ABA biosynthesis and signaling transduction. Additionally, the expression level of *ABI5*, a downstream target of *ABI3*, was also elevated in *ldl1 ldl2* mutants, confirming the effect of LDL1 and LDL2 in ABA signaling transduction. Genetic analysis indicated that mutation of either *aba2* or *abi3* in *ldl1 ldl2* background resulted in reduced seed dormancy compared with *ldl1 ldl2*, suggesting that *ABA2* and *ABI3* are required for LDL1 and LDL2 repressed seed dormancy. Since endogenous ABA level changes also affect the expression level of *ABI3* (Zhang et al., 2005), the increased expression level of *ABI3* and *ABI5* may be pronounced by elevated *ABA2* transcription level in *ldl1 ldl2*. Further study is required to determine whether these genes are direct targets of LDL1 and LDL2 in maturating seeds.

DOG1 was recently identified as a major regulator of seed dormancy independent of ABA in *Arabidopsis thaliana* (Nakabayashi et al., 2012). The protein level of DOG1 in freshly harvested seeds highly correlate with dormancy. The transcription and protein levels of DOG1 gradually increase with the seed maturation (Nakabayashi et al., 2012). In the present study, we found that the transcription level of *DOG1* was significantly up-regulated in the *ldl1 ldl2* mutant, indicating LDL1 and LDL2 regulate *DOG1* expression. Furthermore, the genetic analysis showed that the triple mutant *ldl1 ldl2 dog1* is completely non-dormant, suggesting that *dog1* mutation is epistatic to *ldl1 ldl2* double mutant in seed dormancy.

Recent studies of natural variation in *Arabidopsis* showed that late flowering is correlated with higher seed dormancy. The late flowering mutants *constans* (*co*) and *flowering locus t* (*ft*) display strong increased seed dormancy (Penfield and Hall, 2009; Debieu et al., 2013). Furthermore, lower temperature during the vegetative phase delays flowering time and causes a large increase in the dormancy of seeds produced later on the plants. It was found that maternal past and current temperature experience are transduced to the *FT* locus in silique phloem. In turn, *FT* controls seed dormancy through inhibition of proanthocyanidin synthesis in fruits, resulting in altered seed coat tannin content (Chen et al., 2014). Similar to *co* and *ft* mutants, we showed that the late flowering mutant *ldl1 ldl2* also displays strong increased seed dormancy. Since LDL1 and LDL2 repress *FLC* expression (Jiang et al., 2007) and *FT* acts downstream of *FLC* (Helliwell et al., 2006) in control of flowering, LDL1 and LDL2 may also regulate seed dormancy through regulating *FLC* and *FT* expression.

Interestingly, mutations of the histone methyltransferase *SUVH4* also lead to increased seed dormancy. The expression levels of *DOG1*, *ABI3*, and *ABI4* were elevated in *suvh4* (*kyp-2*) mutant in maturation seeds (Zheng et al., 2012). In present work, an increase of expression of *DOG1* and *ABI3* was detected in *ldl1 ldl2* mutant, whereas the transcription of *ABI4* was not significantly altered. Further research is required to determine whether LDL1, LDL2, and SUVH4 repress seed dormancy through regulating the same target genes such as *DOG1* and *ABI3*.

## Materials and methods

### Plant materials

*Arabidopsis* ecotype Col-0 was used in all experiments, the *abi3-sk11* (SALK_023411) (Park et al., 2011), *ldl1* (SALK_034869), *ldl2* (SALK_135831), *dog1-3* (SALK_000867), and *aba2-1* (CS156), which are in the Col background, were obtained from Nottingham Arabidopsis Stock Centre (NASC). The *ldl1 ldl2* double mutant was generated by crossing *ldl1* with *ldl2*, and the *ldl1 ldl2 dog1-3*, *ldl1 ldl2 abi3-sk11*, and *ldl 1ldl2 aba2-1* triple mutants were generated by crossing *dog1-3*, *abi3-sk11*, and *aba2-1* plants with *ldl1 ldl2* plants, respevtively. For generation of the LDL1 or LDL2 overexpression lines, the full-length open reading frame (ORF) of *LDL1* or *LDL2* was subcloned to pCAMBIA1302 vector under the control of the CaMV 35S promoter with specific primers (Supplemental Table 1), then these constructs were transformed to Col plants following the floral dip assay (Clough and Bent, 1998). The T3 homozygous transgenic plants were used for phenotypic analysis. All the *Arabidopsis* plants were grown at 22°C under long-day (16 h light/8 h dark) conditions. To reduce variations, all genotypes tested in each experiment were grown together and harvested at the same time when most siliques turn brown.

### Subcellular localization analysis

The coding sequences of *LDL1* and *LDL2* without the stop codon were amplified by PCR primers (listed in Supplemental Table 1) and then subcloned into the pSAT6-EYFP-N1 vector and fused in-frame with the Yellow Fluorescent Protein (YFP) sequence under the control of the CaMV 35S promoter. The fusion constructs were introduced into *Arabidopsis* protoplasts by using 40% polyethylene glycol (PEG) as described previously (Yoo et al., 2007). YFP fluorescence was observed with a laser scan confocal microscope (Leica TCS SP2, Leica Microsystems, Wetzlar, Germany). The transient expression assay was repeated three times.

### Germination assay

For the seed dormancy analysis, to make sure that all freshly harvested seeds mature at the same time, we carefully selected plants with siliques that matured at the same time. For the time course of after-ripening germination assay, the seeds or siliques were directly sown on 1/2 MS medium without stratification. For the ABA sensitivity germination assay, only seeds that matured at the same time were selected. After 2 weeks of after-ripening, seeds were sown on 1/2 MS medium supplemented with or without ABA, and then incubated at 4°C for 4 d for stratification or without stratification. The seeds were then placed in a growth chamber at 22°C under long day conditions. Seeds were counted as germinated when the radicle tip had fully penetrated the seed coat (radicle protrusion), and germinated seeds were scored at the indicated times. The statistical analyses were performed with three biological replicates.

### RNA isolation and real-time PCR analysis

Total RNA was isolated from developing siliques (20 siliques) or germination seeds (0.2 g) using 1 mL extraction buffer [0.1 M Tris-HCl, pH 8.0, 0.05 M ethylenediaminetetraacetic acid (EDTA) (pH 8.0), 2% (wt/vol) hexadecyltrimethylammonium bromide (CTAB), 2% (wt/vol) polyvinylpyrrolidone (PVP), 2 M NaCl, 3% β-mercaptoethanol (vol/vol)]. The first strand cDNA synthesis was generated using 2 μg total RNA according to the manufacturer's instructions of TransScript™ One-Step gDNA Removal and cDNA Synthesis SuperMix Kit (TransGen, Beijing). 100 ng synthesized cDNA was used as a template to perform real-time RT-PCR analysis. PCR reactions were performed in the total volume of 20 μL, with 0.5 μL for each primer (10 mM, final concentration 100 nM) and 10 μL for SYBR® Green PCR Supermix (Bio-Rad Laboratories) on a ABI7500 Real-Time PCR System (Applied Biosystems). The PCR program included an initial denaturation step at 94°C for 3 min, followed by 40 cycles of 5 s at 94°C and 1 min at 60°C. Each sample was quantified at least triplicate and normalized using *Ubiquitin 10* (*UBQ*) as an internal control. The gene-specific primer pairs for quantitative Real-Time PCR were listed in Supplemental Table 1. All PCR reactions were normalized using Ct value corresponding to the reference gene *UBQ*. The relative expression levels of target genes were calculated with formula 2^−ddCt^ (Livak and Schmittgen, 2001). Values represented the average of three biological replicates.

### Conflict of interest statement

The authors declare that the research was conducted in the absence of any commercial or financial relationships that could be construed as a potential conflict of interest.

## Supplementary material

Supplementary Figure 1 **Expression patterns of *LDL1* (left) and *LDL2* (right) through the public *Arabidopsis* microarray database (http://www.bar.utoronto.ca/efp/cgi-bin/efpWeb.cgi)**.
