# Supplementary figures and images for: Arabidopsis histone demethylases LDL1 and LDL2 control primary seed dormancy by regulating DELAY OF GERMINATION 1 and ABA signaling-related genes
Source: Front Plant Sci. 2015 Mar 17;6:159. doi: 10.3389/fpls.2015.00159 (PMC4362078; doi:10.3389/fpls.2015.00159)

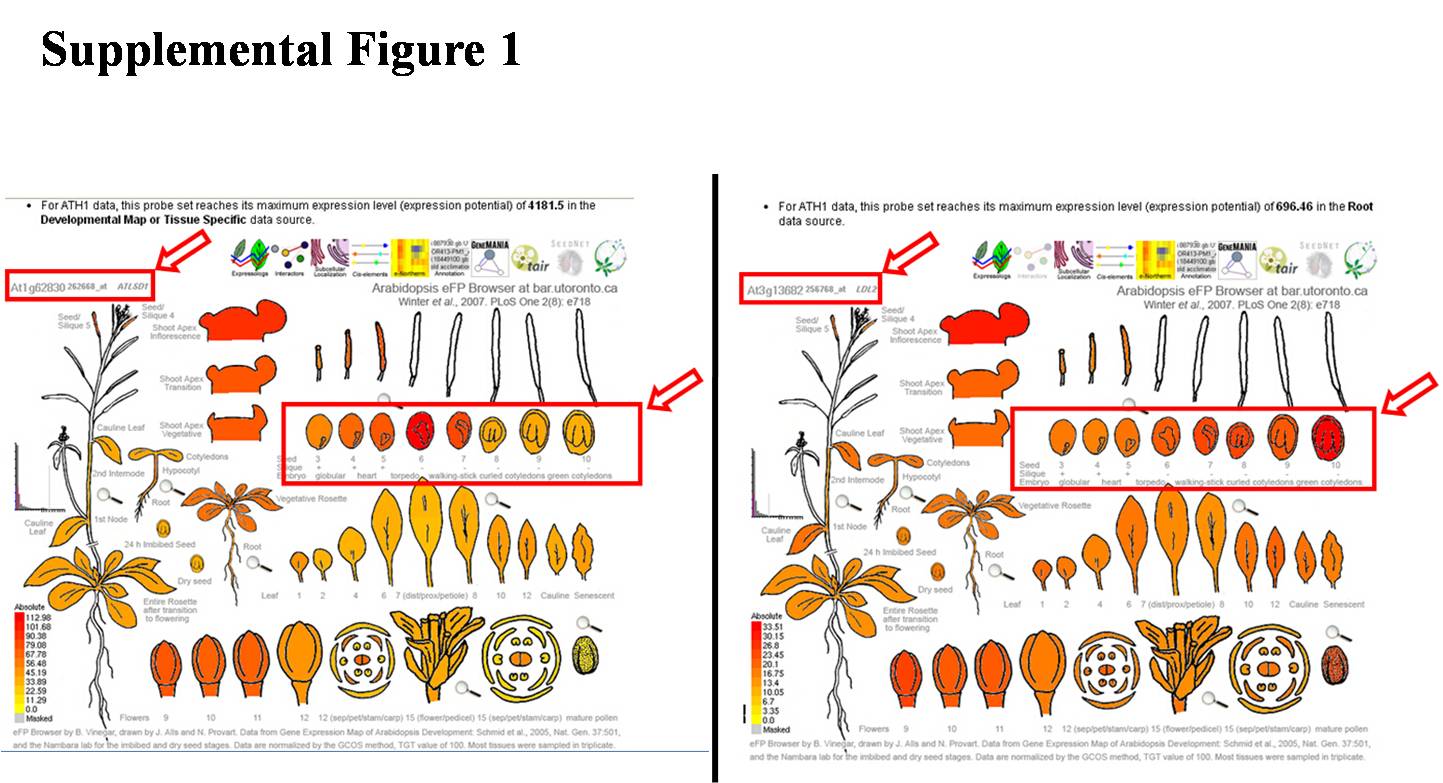

Supplement: Supplementary Figure 1 — Expression patterns of LDL1 (left) and LDL2 (right) through the public Arabidopsis microarray database (http://www.bar.utoronto.ca/efp/cgi-bin/efpWeb.cgi). [file Image1.JPEG]
