# Supplementary material for: Arabidopsis histone demethylases LDL1 and LDL2 control primary seed dormancy by regulating DELAY OF GERMINATION 1 and ABA signaling-related genes
Source: Front Plant Sci. 2015 Mar 17;6:159. doi: 10.3389/fpls.2015.00159 (PMC4362078; doi:10.3389/fpls.2015.00159)
Supplement: Supplementary file 2 [file Table1.PDF]

Supplemental Table 1. Primers used in this study

Name	Sequence(5'-3')	Purpose
LDL1-A	ATGTCAACAGAGACTAAAGAAACCCGACCCG	Genotyping PCR
LDL1-B	ATCAAAGATCTGTCGATTCAGTCTTGCAGC	Genotyping PCR
LDL2-C	ATGAATTCTCCGGCGTCGGATGAAACG	Genotyping PCR
LDL2-D	ATTAAAATGCAGGGGGTTTAAGGGGAGG	Genotyping PCR
DOG1-LP	TTCCAGGAACGTTGTCGTATC	Genotyping PCR
DOG1-RP	AGTTTGTGACCCACACAAAGC	Genotyping PCR
LDL1-YFP-For	CTCAAGCTTCGAATTCATGTCAACAGAGACTAAAGAAACCCG	LDL1 fuse to YFP
LDL1-YFP-Rev	CACCATCAGGATCCCGGGATCAAAGATCTGTCGATTCAGTCTT	LDL1 fuse to YFP
LDL2-YFP-For	CTCAAGCTTCGAATTCATGAATTCTCCGGCGTCGGAT	LDL2 fuse to YFP
LDL2-YFP-Rev	CACCATCAGGATCCCGGGATTAAAATGCAGGGGGTTTAAGGG	LDL2 fuse to YFP
LDL1-qPCR-For	GTCATTGTTGAGGGTTAGGA	qPCR
LDL1-qPCR-Rev	AGTCCTAACTTCTCACGCAA	qPCR
LDL2-qPCR-For	GCTAATGGGTGCCAACTCAG	qPCR
LDL2-qPCR-Rev	TAAGGGGAGGTTACATTGCC	qPCR
LDL1-GFP-For	GGACTCTTGACCATGGTAATGTCAACAGAGACTAAAGAAACCC	LDL1 fuse to GFP
LDL1-GFP-Rev	GTCAGATCTACCATGGTATCAAAGATCTGTCGATTCAGTCTT	LDL1 fuse to GFP
LDL2-GFP-For	GGACTCTTGACCATGGTAATGAATTCTCCGGCGTCGGAT	LDL2 fuse to GFP
LDL1-GFP-Rev	GTCAGATCTACCATGGTATTAAAATGCAGGGGGTTTAAGGG	LDL2 fuse to GFP
ABA1-qPCR-For	GATGCAGCCAAATATGGGTCAAGG	qPCR
ABA1-qPCR-Rev	GCCATTGCATGGATAATAGCGACTC	qPCR
ABA2-qPCR-For	AGAGGTGTTTGCATGATTCCTGAGC	qPCR
ABA2-qPCR-Rev	TCCAGTGATCAATGCCACTTTACCC	qPCR
ABA3-qPCR-For	CAAGCTTGTGTGTATTATCGTCT	qPCR
ABA3-qPCR-Rev	TATACAGGTCCAGTAACAGATTTG	qPCR
AAO3-qPCR-For	AGAGCAGTTGTGAAGCCGTTAG	qPCR
AAO3-qPCR-Rev	ATACTGACCATACGCTTGTTGAAT	qPCR
NCED3-qPCR-For	AACTTCCGTCAAGGGTTCCGTA	qPCR
NCED3-qPCR-Rev	AGCATCCCACTGGTAAATCTCG	qPCR
NCED6-qPCR-For	GGTCGGATATAAATTGGGTTG	qPCR
NCED6-qPCR-Rev	CGGGTTGGTTCTCCTGATTC	qPCR
NCED9-qPCR-For	AACCGCCGCTATGGTTTTAGACG	qPCR
NCED9-qPCR-Rev	CCAGTCACCGGAAGGTTATGCAC	qPCR
ABI3-qPCR-For	CTTGAAGCAAAGCGACGTGG	qPCR
ABI3-qPCR-Rev	TGTCTTACTTTAACCCCTCGTAT	qPCR
ABI4-qPCR-For	TCCGCTCAACGCAAACG	qPCR
ABI4-qPCR-Rev	TTGTCGAACGCCACGGTA	qPCR
ABI5-qPCR-For	CAATAAGAGAGGGATAGCGAACGAG	qPCR
ABI5-qPCR-Rev	CGTCCATTGCTGTCTCCTCCA	qPCR
